# Transient early increase in echocardiographically estimated pulmonary artery pressure after closure of a large patent ductus arteriosus in an adult: a case report

**DOI:** 10.3389/fmed.2026.1854090

**Published:** 2026-08-25

**Authors:** Peng Dai, Rui Xu, Yifan Yan, ZiHan Liang, Zongwei Xiao

**Affiliations:** 1 Department of Cardio-Thoracic Surgery, North Sichuan Medical College, School of Clinical Medicine, Nanchong, Sichuan, China; 2 West China School of Medicine, Sichuan University, Sichuan University Affiliated Chengdu Second People's Hospital, Chengdu Second People's Hospital, Chengdu, Sichuan, China; 3 Department of Cardio-Thoracic Surgery, West China School of Medicine, Sichuan University, Sichuan University Affiliated Chengdu Second People's Hospital, Chengdu Second People's Hospital, Chengdu, Sichuan, China; 4 Department of Clinical Medicine, Henan Medical University, Xinxiang, Henan, China

**Keywords:** adult congenital heart disease, case report, echocardiography, patent ductus arteriosus, pulmonary artery pressure, surgical closure

## Abstract

Pulmonary artery pressure generally decreases after closure of a patent ductus arteriosus (PDA) following elimination of abnormal left-to-right shunting. However, transient increases in echocardiographically estimated pulmonary artery pressure may occasionally occur during the early postoperative period. We report a 41-year-old woman with a large tubular PDA and echocardiographic evidence of pulmonary hypertension. Preoperative computed tomography angiography demonstrated a PDA measuring approximately 13 mm in diameter and 17 mm in length. Echocardiography showed predominantly left-to-right shunting, left atrial and left ventricular enlargement, and an estimated systolic pulmonary artery pressure (SPAP) of 63 mmHg. Right heart catheterization demonstrated a pulmonary vascular resistance of 1.25 Wood units, a transpulmonary gradient of 14 mmHg, and a pulmonary artery wedge pressure of 23 mmHg, findings that argued against advanced fixed pulmonary vascular obstruction and suggested an important high-flow/post-capillary component. After surgical PDA closure, echocardiographically estimated SPAP transiently increased to 101 mmHg on postoperative day 3, accompanied by right ventricular enlargement and reduced left ventricular dimensions. Estimated SPAP subsequently decreased to 60 mmHg on postoperative day 8 and to 38 mmHg at 1 month, with normalization of right-heart dimensions and marked clinical improvement. Diuretics and macitentan were administered during the postoperative period; however, the relative contributions of medical therapy, changes in volume status, postoperative cardiovascular adaptation, and spontaneous recovery cannot be determined from this single case. Because postoperative invasive hemodynamic reassessment was unavailable, the mechanism underlying the transient increase in estimated SPAP remains uncertain. This case illustrates that a marked early rise in echocardiographically estimated SPAP after PDA closure may be transient and should be interpreted in conjunction with serial echocardiographic findings, clinical status, and available hemodynamic data.

## 1 Introduction

Patent ductus arteriosus (PDA) is a congenital cardiovascular lesion characterized by persistent communication between the descending aorta and pulmonary artery (1). In hemodynamically significant PDA, chronic left-to-right shunting may result in increased pulmonary blood flow, left-heart volume overload, and varying degrees of pulmonary artery pressure elevation (1, 2). In adults with a significant PDA and acceptable pulmonary vascular resistance, closure is generally recommended, with transcatheter closure preferred when anatomically feasible and surgical closure considered in patients with unfavorable or complex ductal anatomy (1, 3–5). In the absence of advanced irreversible pulmonary vascular obstructive disease, elimination of the shunt is generally expected to reduce pulmonary overcirculation and pulmonary artery pressure (6).

However, transient increases in echocardiographically estimated pulmonary artery pressure may occasionally be observed during the early period after PDA closure (6, 7). The interpretation of such findings can be challenging, particularly when postoperative invasive hemodynamic measurements are unavailable. An early increase in estimated pulmonary artery pressure may reflect several interacting factors, including abrupt changes in loading conditions, postoperative volume status, ventricular interaction, or changes in pulmonary vascular tone. Distinguishing transient postoperative adaptation from persistent pulmonary vascular disease is therefore clinically important.

**Figure 1:**
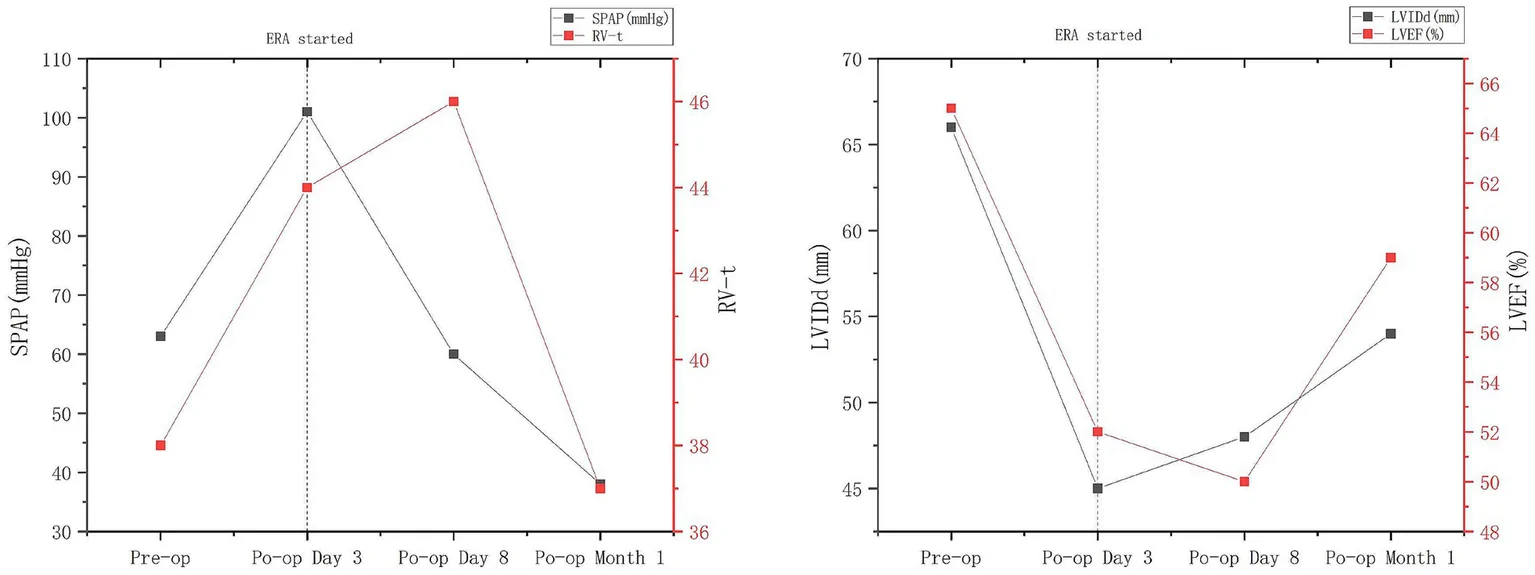
Echocardiographic data change chart.

We report the case of a 41-year-old woman with a large tubular PDA who developed a marked but transient early increase in echocardiographically estimated SPAP after surgical closure, despite a low preoperative PVR. Estimated SPAP increased from 63 mmHg before surgery to 101 mmHg on postoperative day 3 and subsequently decreased to 38 mmHg at 1 month, accompanied by normalization of right-heart dimensions and clinical improvement. This case highlights the importance of cautious interpretation of early postoperative echocardiographic changes after PDA closure, particularly when invasive postoperative hemodynamic reassessment is unavailable. The temporal evolution of this case also illustrates the potential reversibility of such postoperative echocardiographic abnormalities (see Figure 1).

## 2 Case presentation

### 2.1 Preoperative

A 41-year-old woman presented with a more than 2-year history of chest tightness and intermittent chest pain radiating to the chest and back, with symptoms worsening during physical activity. Bilateral lower-extremity edema was also noted. Her symptoms had gradually progressed, and reduced exercise tolerance had developed approximately 2 months before surgery. She had no history of hypertension, connective tissue disease, or deep vein thrombosis. Physical examination revealed a continuous machinery murmur with a palpable thrill at the left sternal border in the third intercostal space.

Transthoracic echocardiography demonstrated a tubular PDA measuring approximately 8 mm in width, with predominantly left-to-right shunting and a minor bidirectional component. Left atrial and left ventricular enlargement were present, with a left ventricular internal dimension at end-diastole (LVIDd) of 66 mm, a right ventricular transverse diameter (RV-t) of 38 mm, an estimated SPAP of 63 mmHg, and a left ventricular ejection fraction (LVEF) of 65%. Computed tomography angiography further demonstrated a large tubular PDA measuring approximately 13 mm in diameter and 17 mm in length. Electrocardiography suggested possible right ventricular hypertrophy. The NT-proBNP level was 760 pg./mL, whereas high-sensitivity cardiac troponin T was within the normal range.

Preoperative right heart catheterization showed a pulmonary artery pressure of 60/37/26 mmHg (systolic/mean/diastolic) and a pulmonary artery wedge pressure of 24/23/23 mmHg, corresponding to a transpulmonary gradient of 14 mmHg and a pulmonary vascular resistance of 1.25 Wood units. The original catheterization report documented a Fick-derived Qp/Qs ratio of approximately 1. However, the complete raw data required for independent verification of the Fick calculation were unavailable. Therefore, the reported Qp/Qs value was interpreted cautiously.

The reported Qp/Qs ratio appeared discordant with the overall clinical findings. Echocardiography demonstrated a large PDA with predominantly left-to-right shunting and marked left-heart enlargement, while oximetry showed an oxygen saturation step-up from approximately 72% in the right ventricle to 87% in the main pulmonary artery, supporting ductal-level left-to-right shunting. Because the complete raw Fick data were unavailable, the reported Qp/Qs ratio could not be independently verified and was therefore not used as a primary basis for subsequent interpretation.

Pulmonary vasoreactivity testing and temporary test occlusion were not performed. Taken together, the large PDA anatomy, predominantly left-to-right shunting, left-heart enlargement, elevated pulmonary artery wedge pressure, and low preoperative PVR were considered consistent with a hemodynamically significant PDA with chronic left-heart volume overload, without evidence of markedly elevated fixed pulmonary vascular resistance. Based on the ductal anatomy and the overall preoperative assessment, surgical closure of the PDA under deep hypothermic circulatory arrest was performed.

### 2.2 Postoperative

#### 2.2.1 Post-op day 3

The patient reported improvement in chest discomfort and shortness of breath compared with her preoperative status and was able to ambulate with assistance. However, echocardiography showed a marked early postoperative increase in estimated SPAP from 63 mmHg preoperatively to 101 mmHg. This was accompanied by right ventricular enlargement (RV-t, 44 mm), a reduction in LVIDd from 66 to 45 mm, and a decrease in LVEF to 52%. Furosemide and macitentan were initiated. Postoperative right heart catheterization was not performed. Although the echocardiographic changes were marked, the patient’s clinical symptoms did not deteriorate correspondingly, and she declined further invasive assessment after informed discussion.

#### 2.2.2 Post-op day 8

The patient’s symptoms of cardiac fatigue and shortness of breath were significantly relieved compared with before surgery, with no complaints of fatigue or shortness of breath during daily activities, and she was discharged. Follow-up echocardiography showed that SPAP had decreased to 60 mmHg, but the right ventricle remained enlarged (RV-t, 46 mm), and LVEF was 50%. Macitentan was continued, and after full informed consent, the patient declined further invasive examination. Considering the significant short-term improvement in symptoms and echocardiographic indicators, the clinical urgency for additional right heart catheterization was reduced.

#### 2.2.3 Post-op month 1

The patient reported no symptoms of cardiac fatigue or shortness of breath, including after walking 600 m. Follow-up echocardiography showed that SPAP had further decreased to 38 mmHg, right-heart dimensions had normalized, and LVEF had recovered to 59%. Macitentan was continued.

#### 2.2.4 Post-op month 2

Telephone follow-up showed that the patient had no obvious clinical symptoms.

## 3 Treatment

### 3.1 Management and rationale

Preoperative right heart catheterization demonstrated a PVR of 1.25 Wood units and a TPG of 14 mmHg, findings that did not suggest markedly elevated fixed pulmonary vascular resistance. In contrast, the pulmonary artery wedge pressure was elevated at 23 mmHg, while echocardiography demonstrated substantial left-heart enlargement associated with the large PDA. These findings suggested that the elevated pulmonary artery pressure occurred in the setting of significant left-heart volume overload and a post-capillary component rather than isolated severe precapillary pulmonary vascular disease. Based on the overall clinical, anatomic, echocardiographic, and catheterization findings, PDA closure was considered appropriate.

Preoperative computed tomography angiography showed a large, wide tubular PDA measuring approximately 13 mm in diameter and 17 mm in length, without definite ductal aneurysm or severe calcification. Because of the broad diameter of the adult ductus, conventional external circumferential dissection and simple ligation were considered to carry an increased risk of injury at the ligation site (1, 5, 8). In a large pressurized ductus, forceful external ligation may concentrate compression and shear stress within a narrow band of tissue, potentially increasing the risk of ductal wall tearing, rupture, or bleeding from the adjacent descending aorta or pulmonary artery (5, 8). Therefore, direct closure of the internal ductal orifice under deep hypothermic circulatory arrest was selected to permit direct visualization of the ductal margins and secure closure in a bloodless operative field without forceful circumferential compression (5, 8). Deep hypothermia was also used to reduce cerebral metabolic demand and provide cerebral protection during temporary circulatory arrest (9, 10).

On postoperative day 3, furosemide and macitentan were initiated in response to the marked increase in estimated SPAP and right ventricular enlargement. Macitentan is a dual endothelin-receptor antagonist used in the treatment of pulmonary arterial hypertension (11, 12). The use of macitentan represented an individualized clinical decision rather than treatment directed at a definitively established postoperative pulmonary vascular mechanism.

Estimated SPAP subsequently decreased and the patient’s clinical status improved. However, this temporal association does not establish a specific treatment effect of macitentan. Concomitant diuretic therapy, changes in volume status, postoperative cardiovascular adaptation, and spontaneous recovery may all have contributed to the observed course.

### 3.2 Follow-up after discharge

At the 1-month outpatient follow-up, the patient remained clinically well, with marked improvement in exercise tolerance and no recurrent fatigue or shortness of breath. Echocardiography demonstrated normalization of right-heart dimensions and a decrease in estimated SPAP to 38 mmHg. Macitentan was continued temporarily under clinical and echocardiographic surveillance. Because the observed improvement may also have reflected diuresis, changes in volume status, postoperative adaptation, and spontaneous recovery, the specific contribution of macitentan could not be determined. At the most recent follow-up, the patient remained clinically stable (see Table 1).

**Table 1:** Serial changes in clinical status and echocardiographic parameters before and after PDA closure.

Time point	SPAP (mmHg)	RV-t (mm)	LVIDd (mm)	LVEF (%)	Main clinical features	Treatment
Pre-op	63	38	66	65	Dyspnea, large PDA with left-to-right shunt	Planned surgical closure
Post-op day 3	101	44	45	52	Acute RV enlargement, LV underfilling	Diuretics + macitentan
Post-op day 8	60	46	48	50	Symptoms improved	Continued therapy
Post-op month 1	38	37	54	59	Marked clinical recovery	Follow-up

## 4 Discussion

In patients with a hemodynamically significant PDA, closure is generally expected to reduce pulmonary overcirculation and pulmonary artery pressure when markedly elevated fixed pulmonary vascular resistance is absent (3, 4, 6). In the present patient, however, echocardiographically estimated SPAP increased from 63 mmHg preoperatively to 101 mmHg on postoperative day 3 before decreasing to 60 mmHg on day 8 and 38 mmHg at 1 month. This marked but transient early postoperative increase in estimated SPAP represents the principal clinical observation of this case. Importantly, the echocardiographic change occurred despite concurrent improvement rather than worsening of the patient’s clinical symptoms.

Preoperative hemodynamic findings are important for interpreting this course. Right heart catheterization showed a PVR of 1.25 Wood units and a TPG of 14 mmHg, which did not suggest markedly elevated fixed pulmonary vascular resistance. At the same time, the PAWP was elevated at 23 mmHg, and echocardiography demonstrated marked left atrial and left ventricular enlargement. Together with the large PDA and predominantly left-to-right shunting, these findings indicate substantial chronic volume overload and an important post-capillary component. Thus, the elevated preoperative pulmonary artery pressure should not be interpreted as evidence of isolated advanced precapillary pulmonary vascular disease (13).

The original catheterization report documented a Fick-derived Qp/Qs ratio of approximately 1. This value was discordant with the large PDA anatomy, predominantly left-to-right shunting, left-heart enlargement, and the observed oxygen saturation step-up. Because the complete raw Fick data was unavailable, the reported Qp/Qs could not be independently verified. It was therefore interpreted cautiously and was not used as a basis for mechanistic interpretation.

The mechanism underlying the transient postoperative increase in estimated SPAP cannot be established from this case. Closure of a large, long-standing left-to-right shunt abruptly alters pulmonary blood flow, ventricular loading conditions, left ventricular preload, and ventricular interaction. In a cardiovascular system chronically adapted to substantial volume overload, such changes may contribute to transient postoperative echocardiographic abnormalities. Changes in postoperative volume status and pulmonary vascular tone may also have played a role (14, 15). However, because postoperative right heart catheterization and pulmonary vasoreactivity testing were not performed, the relative contributions of these factors cannot be determined.

The subsequent course supports the transient nature of the postoperative echocardiographic changes. Estimated SPAP progressively decreased over the following weeks, accompanied by normalization of right-heart dimensions, partial recovery of left ventricular dimensions and systolic function, and sustained symptomatic improvement. This temporal pattern argues against interpreting the isolated early postoperative echocardiographic finding as evidence, by itself, of irreversible progression of pulmonary vascular disease. Serial assessment of clinical status and echocardiographic findings may therefore be particularly important when marked early postoperative changes are observed (16).

Macitentan and diuretic therapy were initiated during the early postoperative period, followed by a reduction in estimated SPAP. However, the contribution of macitentan cannot be isolated from concomitant diuresis, changes in volume status, spontaneous postoperative cardiovascular adaptation, and natural recovery. Accordingly, the clinical course should not be interpreted as evidence of a specific endothelin-mediated mechanism or a specific therapeutic effect of macitentan.

Several limitations should be acknowledged. First, postoperative right heart catheterization was not performed; therefore, the marked increase in echocardiographically estimated SPAP could not be confirmed by invasive pulmonary artery pressure measurement, and postoperative changes in PAWP, pulmonary blood flow, and PVR could not be quantified. Second, pulmonary vasoreactivity testing was not performed, limiting assessment of changes in pulmonary vascular tone. Third, the preoperative Fick-derived Qp/Qs ratio could not be independently verified because the complete raw calculation data were unavailable. Finally, this is a single-case observation, and the temporal association between medical therapy and subsequent improvement cannot establish causality. These limitations preclude definitive conclusions regarding the mechanism of the postoperative echocardiographic changes.

## 5 Conclusion

This case demonstrates that a marked early increase in echocardiographically estimated SPAP may occur transiently after closure of a large PDA, even in a patient with low preoperative PVR. Estimated SPAP increased from 63 mmHg before surgery to 101 mmHg on postoperative day 3 and subsequently decreased to 38 mmHg at 1 month, accompanied by normalization of right-heart dimensions and clinical improvement. Because postoperative invasive hemodynamic reassessment was unavailable, the mechanism underlying these changes could not be determined. An early postoperative increase in estimated SPAP should therefore be interpreted in conjunction with serial echocardiographic findings, clinical status, and available hemodynamic data rather than being considered, in isolation, evidence of irreversible pulmonary vascular deterioration.

## Data Availability

The data analyzed in this study is subject to the following licenses/restrictions: Data will be available on request from corresponding author. Requests to access these datasets should be directed to ZX, 734269726@qq.com.
